# Evaluation of thermal performance factor by hybrid nanofluid and twisted tape inserts in heat exchanger

**DOI:** 10.1016/j.heliyon.2022.e11950

**Published:** 2022-11-30

**Authors:** Noor Fouad. A. Hamza, Sattar Aljabair

**Affiliations:** Mechanical Engineering Department, University of Technology, Iraq

**Keywords:** Hybrid nanofluids, Double V-cut twisted tape, Heat transfer enhancement, CFD, Thermal performance factor

## Abstract

The thermal performance parameters of an improved heat exchanger tube fitted with various vortex generator inserts were investigated using numerical and experimental methods. The governing equations have been solved numerically by a Finite Volume approach employing the turbulence model (κ−ε). Two twisted tape types, which being inserted across a circular pipe (plain twisted tape) and (Double V-cut twisted tape), have been achieved. The hybrid nanofluid is prepared by using metal oxide [Al_2_O_3_+CuO] with distilled water at volume fraction range (0.6%, 1.2% and 1.8%), Reynolds number range (3560–8320) at twisted ratio (9.25). The experimental data for a plain tube, plain twisted tapes and double v-cut twisted tape are validated using the standard correlations available in the literature. The effect of such variables upon the average Nusselt number, friction factor, and thermal performance factor have been investigated and compared with a plain tube at the same conditions. As compared to plain twisted tape, the tube equipped with a double V-cut twisted tape with hybrid nanofluid displayed increased thermal performance. The greater vortex flow induced by the V-cuts results in more active thermal boundary layer disturbance, resulting in a greater heat transfer rate. The results show that thermal performance factor for hybrid nanofluid in plain circular tube at (∅=1.8%) and Reynolds number (8320) is about (1.068), when the plain twisted tape and double v-cut twisted tape inserted with hybrid nanofluid the thermal performance factor increased to (1.33) and (1.37), respectively. The results show a similar trend for both numerical and experimental cases. The comparison between the experimental and numerical results have maximum error was (9.7)%.

## 1 Introduction

Heat exchangers are widely used for energy conversion and recovery in various industrial, social, and commercial sectors. The efficiency of a heat exchanger is reduced because the heat exchange between the solid and the fluid is relatively low due to the high thermal resistance provided by the thermal boundary layer at the solid–fluid contact. Several strategies have been implemented to minimize this problem, including:

• Modification the surface pipe wall [1], [2].
• Employing nanofluid as a working fluid [3].
• Incorporating swirls into internal flow [4].

**Figure 1:**
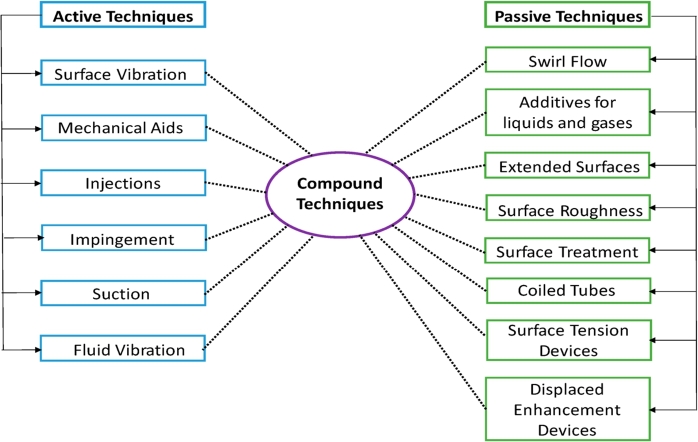
Categorization of various techniques for heat transfer enhancement [5].

Also heat transfer enhancement techniques classified as shown in Fig. 1.

Nanofluids were developed in the last century to keep up with the ongoing increase in the efficiency of thermal systems. Nanoparticles of materials with high thermal conductivity, such as metals, metal oxides, copper, and carbon nanotubes (size diameter less than 100 nm) were applied to base fluid to increase the efficient thermal conductivity. A Nanofluids is a substance that contains nanoparticles, which are particles that are smaller than a nanometer. Several studies have reported using Nanofluids to improve heat transfer [6], [7], [8].

Hybrid nanofluids are a relatively new type of nanofluids with several potential uses in heat transfer e.g. manufacturing, transportation, defense, medical, naval structures, and acoustics, among other fields. The phrase hybrid nanofluids refers to a suspension of multi nanoparticles types in a base fluid that's thought to increase thermo-physical qualities whilst introducing them in a highly steady way via combine the desired chemical and physical properties of the material [9], [10]. There are two phases in this field, single-phase, two-phase models are used to quantitatively study the heat transport of nanofluids. Hussein [11] used MWCNT nanofluids and MWCNTs/GNPs hybrid nanofluids were tested for thermal performance with a Reynolds number from 200 to 500. For different weight fraction of MWCNTs (from 0.075 to 0.25 wt.%), a theoretical model was used to predict the thermal conductivity of MWCNTs-water nanofluids. The viscosity of the nanoparticle concentrations increased slightly using MWCNTs nanofluids and MWCNTs/GNPs hybrid nanofluids, so the Nusselt number was improved. Where the heat transfer coefficient is proportional to the concentration of nanoparticles and inversely proportional to Reynolds number. Further, using nanofluids resulted in an 11% increase in pressure decrease. The maximum enhancement was 43.4% at Re = 200. Also, thermophysical characteristics and the fundamental function of Brownian force are responsible for this improvement. Additionally, while utilizing nanofluid, a pressure drop increase of 11% was observed. Thus, the weight concentrations of MWCNTs determine how adding GNPs affects pressure decrease.

Twisted tape inserts are one of the widely used methods for generating turbulence close to the wall because they produce an internal swirl flow that lengthens the pipeline's fluid flow direction, improved fluid mixing, and reduced area of thermal boundary, that way increasing the rate of convection heat transfer [12], Kumar [13] dispersed hybrid nanofluids, water was mixed with various nanoparticle mixtures. They thought of nanoparticles: Al_2_O_3_, MgO, SiC, AlN, MWCNT, and Cu, in various variations such as oxide-metal, oxide-nitride, oxide-carbide, and oxide-carbon nanotubes with an equivalent ratio of (0.1, and 0.5) L/m for laminar regime levels, fluid temperatures (20 °C, and 40 °C), and (50) W/cm^2^ for heat flux. The findings revealed that there was an increase in flow rate and a decrease in thermal resistance, as well as an increase in heat transmission coefficient. This form of insertion has been studied of different previous researchers, twisted tapes [14], [15], [16], [17], helical screw [18], [19], [20], wire coil [21], [22] and winglet [23], [24].

Many studies have investigated passive ways to increase the heat exchanger's performance. [25] addressed the heat transfer in a double pipe heat exchanger with an orderly and spaced twisted tape was tested experimentally. The inner and outer diameters of the pipe are (25.8) mm and (50.6) mm, respectively, by using hot and cold water on the tube side and shell side. The twisted tape was made from stainless steel of (1500) mm with length, (1) mm thickness and twisted ratio (6.0 and 8.0) and many free space ratios (1.0, 2.0, and 3.0). The results show an increase in heat rate with twist ratio while the friction factor and heat transfer coefficient will modulate by increasing the free space ratio.

Karamallah et al., [26] studied numerical and experimental horizontal pipes for the turbulent flow and constant heat flux. In a single-phase, three types of nanofluid are used (Al_2_O_3_, ZrO_2_, and CuO) were mixed with distilled water, twist ratios (TR = 4, 6, 8), and three different twisted tapes geometries were used (twisted with V- cut, typical type and clockwise-counter clockwise type). Nanoparticle concentrations of (0, 0.1, 0.05, 0.1, 0.5, 1, 2, 3) % for Reynolds number range (from 2490 to 20100) and heat flux (from 2108 to 9280) W/m^2^. The results present the CuO with clockwise or counter clockwise, twisted tape equal (TR = 4), Reynolds number value (2490) and volume fraction (∅=3%) gives the maximum augmentation in heat transfer rate close to (7.5 times Nu number in water) and (TPF = 3.9) for CuO with clockwise-counter clockwise twisted tape and twist ratio equal (4) at Reynolds number (2490) and (∅=3%). Singh et al., [27] studied the effects of tapered coils in double pipes on the thermo-physical properties of 50 nm Al_2_O_3_ and 90 nm MgO with water over as a (base fluid) over a Reynold number scale of 9,000 to 40,000 at nanofluids inlet temperature (50 C, 60 C and 70 C). The researchers used wire coils of aluminum with one end 13 mm in diameter, the other 6.5 mm in diameter and a permanent pitch of 10 mm for the convergence, divergence, and convergence-divergence types. It was discovered that as the Re number increased, Nu and the fraction factor decline increased leading to a reduction in the thickness of both momentum and thermal boundary layers. Furthermore, the long path flow increased by friction factor. The analysis showed a significant increase in heat transfer in the divergence style wire coil due to the strong contact area between nanofluids and tube wall. Hasanpour et al., [28] investigated the heat transfer rate and the pressure losses in a corrugated tube heat exchanger with several kinds of twisted tapes using both experimental as well as numerical methods. It was discovered that the TPF of twisted tapes having V-cut is always greater than one, with a maximum value of (1.50). Hosseinnezhad et al., [29] conducted a numerical study for turbulent case of water/Al_2_O_3_ nanofluids in a tubular heat exchanger with double twisted tape inserts. Case study applied for Reynolds numbers range (10000–30000), twist ratio from (2.5 to 4), co-swirl flow and counter-swirl flow, two twisted tapes, and volume concentration of nanoparticle's range (1, 4) percent. The findings of this study revealed that increasing the average Nusselt number by lowering the twist ratio, counter-swirl flow twisted-tape, and increasing the volume concentration of Al_3_O_2_ nanoparticles in the base fluid. In twist ratios of (3.25) and (2.5), the maximum amounts of performance evaluation criterion (PEC) were (1.6) and (1.55) respectively. Therefore, the maximum value of PEC in three cases of twist ratios (4, 3.25, and 2.5) were (40.8%, 47%, and 51%) respectively. While the maximum e value of PEC in the three mentioned ratios were (26.5%, 28.3% and 30.6%) respectively. Dalkılıç et al., [30] studied the experimental investigation of turbulent heat transfer properties of Graphite-SiO_2_-Water hybrid nanofluids flow in a horizontal tube with and without quad-channel twisted tape (QCTT) inserts. The hybrid nanofluids was made up of two types of nanoparticles: silicon dioxide (60%) and graphite (40%) with water as the base fluid. Experiments were carried out at two distinct volume concentrations (0.5% and 1%) and Reynolds number ranges (3400 - 11000). According to the results, the Nusselt number of the hybrid nanofluids, mass flow rate and volume fraction increased. In addition, as the length of the twisted tape insert was extended, the heat transfer coefficient increased. Furthermore, with rising Reynolds number, friction factor increases with increasing volume concentration, but friction factor increases with increasing tape insert length. As the mass flow rate and volume concentration rise, the pressure drop increases. [31] Entropy generation in this study is very important in our project and we will study that in the next step of the research.

Finally, the regression equations are shown to be well-matched with the experimental results within a ∓5% and ∓10% deviation zone for the Nusselt number and friction factor, respectively.

Many important kinds of research are surveyed with any information about the techniques of heat transfer enhancement as shown in Table 1, it is apparent that the simultaneous use of nanofluids, hybrid nanofluids, and twisted tape effectively further improves the heat transfer enhancement with respect to the individual use of twisted tape or hybrid nanofluids.

**Table 1:** Summery the previous studies.

Ref.	Geometry	Study	Working fluid	Volume concentration	Reynolds number range	Passive technique	Improvement
[10]	Circular tube with constant heat flux	Exp.	(TiO_2_–SiO_2_/ Water + Ethylene glycol	(∅ = 1%)	Re 200-500	—–	Thermal performance increases by 35.3%, while the friction factor also increases compared to the base fluid.
[11]	Tube with square twisted tape	Num.	(water)	mass flow rate equal 0.1027 kg/s	Re 8000-18000	y/w = 2.7, 4.5, 6.5	• Classical twisted tape and square twisted tape have higher enhancement in heat transfer. • Square twisted tape with y/w = 2.7 has higher heat transfer enhancement.
[12]	Mini channel heat sink	Exp.	Al_2_O_3_, MgO, SiC, AlN, MWCNT, and Cu)/ Water	(0.01 vol%)	Re 50-500	—–	Increase in flow rate and a decrease in thermal resistance, as well as an increase in heat transfer coefficient.
[26]	Double tube heat exchanger	Exp.	(Al_2_O_3_, MgO)/ water	0.1% volume concentration	Re 9000-40000	(d = D/2) from another end	Re number increased, Nu and the fraction factor decline increased due to the decrease in both momentum and thermal boundary layer thicknesses
[28]	Tube	Num.	water/Al_2_O_3_	1 to 4%	Re 10000-30000	Co-swirl and counter-swirl flow of two twisted-tapes TR = 2.5 to 4	• The max. enhancement equal 5.19% at TR (2.5) and Re of (30000). • The min. enhancement equal 1.29% at TR (4) and Re of (10000).
[29]	Horizontal tube	Exp.	(Graphite-SiO_2_)+ water Hybrid nanofluids	0.5% and 1%	Re 3400-1000	Quad-channel twisted tape	increasing mass flow rate and volume fraction, and twisted tape length leads to increase heat transfer rate.
Present work	horizontal tube	Num. + Exp.	Al_2_O_3_+ CuO /Water Hybrid nanofluids	(∅ = 0.6,1.2 and 1.8)% by volume	Re 3560-8230	TR = 9.25 used PTT, DVCT	• Nu for DVCT with hybrid nanofluids more than Nu for PTT • Thermal performance for DVCT higher than PTT

The aims of this study investigate the effects of new combination of twisted tape fitted in heat exchangers tube with hybrid nanofluids (Al_2_O_3_, CuO/distilled water) under turbulent flow operation, where twisted tape insert used as a turbulator and hybrid nanofluids as a working fluid for varied geometries, it was reported on how this combination improves the thermal performance factor and heat transfer rate of the heat exchanger.

## 2 Methodology

**Figure 2:**
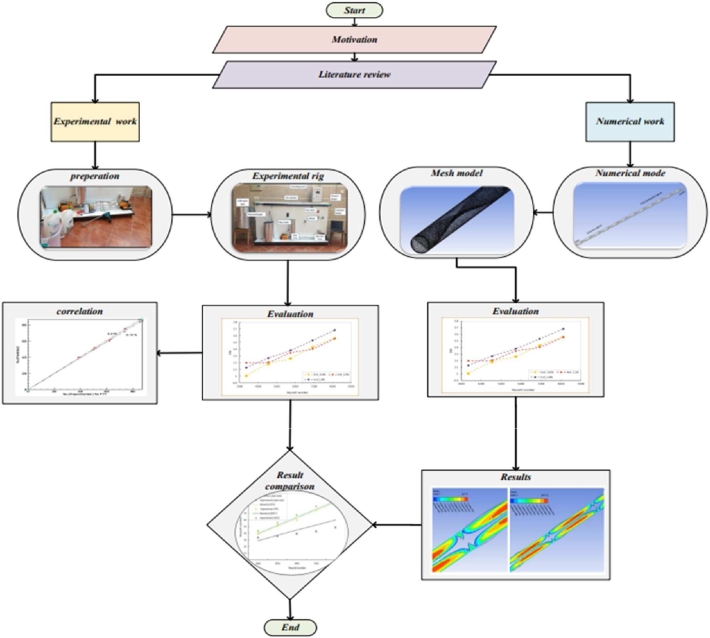
Main steps of the present study.

The creation of the model geometries and its integration in a physical domain, grid generation, and the choice of an appropriate numerical computing technique are all significant factors that may influence the success of the simulation process. The complicated process of heat transfers and fluid movement into the heat pipe is further complicated by the creation of the model geometries and its integration in a physical domain. On a smooth horizontal tube with twisted tapes and a hybrid nanofluid, turbulent flow experiments and numerical simulations were conducted, and the results were validated. This quantitative study adopted five values of Reynolds numbers ranging from (3560 - 8320) under constant heat flux (13217.4 W/m^2^). The main steps of the performed studies are briefly described in the following paragraphs, as shown in Fig. 2.

## 3 Experimental procedure

**Figure 3:**
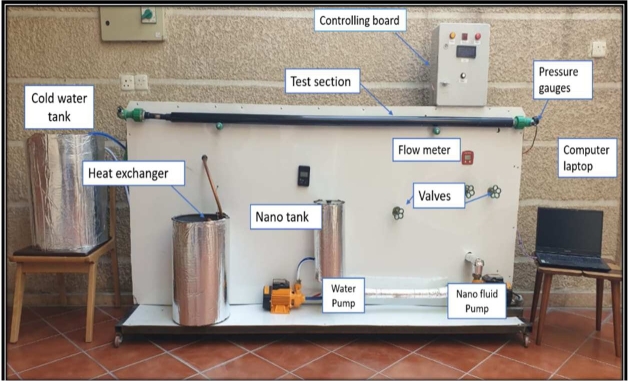
Photographic presentation of experimental setup.

The experimental test rig was designed; Photographic presentation of experimental setup is shown in Fig. 3. It is primarily made up of copper tube, fabricated twisted tapes, hybrid nanofluids tank, cooling fluid cycle (helical heat exchanger), and control panel as shown below.

**Figure 4:**
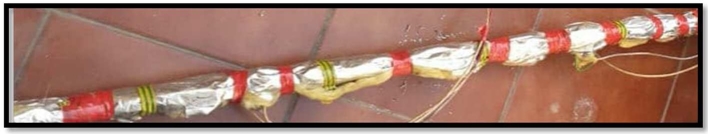
Copper tube.
II. **Twisted tapes:** Three-dimension printer devise was used to fabricate the (Plain twisted tape and Double V- cut tape). PLA (poly lactic acid) was employed in the manufacturing process, as shown in Fig. 5. The twisted tape criteria are the ratio of the width of the twisted tape to the tube diameter must be less than (1), with the twist tape thickness and twist ratio being interdependent for optimum design.

I. **Copper tube:** The copper tube used for the circular working section had a (0.02) m diameter and a (2) m length. It is heated by a tungsten-based, eight-meter-long electrical coil with a 2000 W heating capacity. By connecting the tube to an AC power source, heat flux is produced. The tube's entrance length when it is fully formed is (1.5 m). The test area was insulated with rock wool insulation. Three thermocouples (K-type), each separated by (50 cm), were used to measure the temperatures along the outside surface of the tube at the heated test section., as shown in Fig. 4.

**Figure 5:**
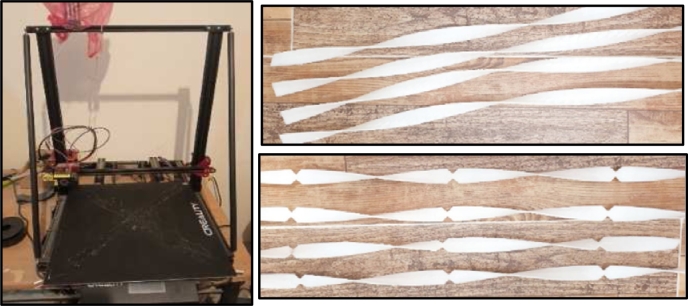
Twisted tape manufacture.
III. **Hybrid nanofluids reservoir**: It is a plastic tank with dimensions of (15 cm) diameter and (40 cm) height it covered with aluminum shell; it represents the feeding source of the hybrid nanofluids to the test rig. The pump is connected to the lowest point in the tank. The valves are adjusted to ensure that the desired flow rate of hybrid nanofluids flows to the test section, and the excess nanofluids is returned to the reservoir through the bypass line.
IV. **Cooling fluid cycle**: To ensure that overheating does not occur, a spiral heat exchanger was fabricated from a (15 m) long and (19 mm) diameter spiral copper coil and used to cool the hot hybridfluid coming from the test section. The coil was placed in a plastic tank of (50 cm) height and (30 cm) diameter filled with re-circulating water. The tank insulated by a glass wool and rubber with thickness (20 mm).
V. **Control panel**: The electric board was used to organize the functioning components and is separated into the two sections (The Electric Section, The Arduino Section).

## 4 Repeatability check

To validate the test rig's performance and the impacts of the environment on the experimental findings, multiple test runs were conducted under specific operating circumstances for the applied heat flux and fluid flow rates. When the results of the test runs were compared, a difference of nearly 3.6% for the temperature readings and ±2 percent for pressure drop values was determined.

## 5 Uncertainty analysis

The parameters required to calculate the Nusselt number are unclear due to measurement equipment uncertainty. The analysis was carried out according to Robert J. Moffat [32] recommendations. The experiment's uncertainties were assessed using the independent parameters measured in the experiments, where Table 2 shows the instrument utilized in this study, along with its inaccuracy and range.

**Table 2:** Nusselt number for hybrid nanofluids for smooth case tube.

No.	Flow rate (L/min)	Error of Nusselt number $h_{nf}$ at ∅=0.6%	
		Error	Relative error
1	3	0.0217	0.00070
2	4	0.0144	0.00040
3	5	0.0098	0.00026
4	6	0.00092	0.00020
5	7	0.00084	0.00017

(R) is calculated as shown:

(1a) $$R=R(X_{1},X_{2}X_{3}\ldots \ldots ..,X_{i})\;\delta RX_{i}=\frac{\partial R}{\partial X_{i}}⁎\delta X_{i}$$

(1b) $$\delta R={\left[\sum_{i=1}^{m}{\left(\frac{\partial R}{\partial X_{i}}⁎\delta X_{i}\right)}^{2}\right]}^{0.5}$$

According to the outcome, the uncertainty interval (S) can be expressed as follows:

(1c) $$S_{R}={\left[{\left(\frac{\partial R}{\partial {\overset{˙}{X}}_{1}}S_{X1}\right)}^{2}+{\left(\frac{\partial R}{\partial {\overset{˙}{X}}_{2}}S_{X2}\right)}^{2}+\ldots \ldots \ldots \ldots +{\left(\frac{\partial R}{\partial {\overset{˙}{X}}_{i}}S_{\mathrm{Xi}}\right)}^{2}\right]}^{0.5}$$

In a dimensionless form:

(1d) $$\frac{S_{R}}{R}={\left[{\left(R_{X1}S_{X1}\right)}^{2}+{\left(R_{X2}S_{X2}\right)}^{2}+\ldots \ldots \ldots \ldots +{\left(R_{\mathrm{Xi}}S_{\mathrm{Xi}}\right)}^{2}\right]}^{0.5},\text{Where, }R_{\mathrm{Xi}}=\frac{\partial R}{\partial {\overset{˙}{X}}_{i}}$$

The Nusselt number:

(1e) $$\mathrm{Nu}=\frac{h_{\mathrm{in}}d_{\mathrm{in}}}{k}=\frac{Qd_{i}}{A_{S}\Delta T_{S}K}=\frac{\overset{˙}{m}C_{P}\Delta T_{b}d_{i}}{A_{S}\Delta T_{S}K}$$

$$\Delta T_{\mathrm{bulk}}=T_{\mathrm{out}}-T_{\mathrm{in}},\;\Delta T_{s}=T_{wall}-T_{bulk},\;A_{S}=\pi d_{o}L$$

For Nusselt number the uncertainty is calculated as below:

(1f) $$S_{\mathrm{Nu}}={\left[{\left(\frac{\partial \mathrm{Nu}}{\partial \overset{˙}{m}}S_{\overset{˙}{m}}\right)}^{2}+{\left(\frac{\partial \mathrm{Nu}}{\partial \Delta T_{b}}S_{\Delta T_{b}}\right)}^{2}+{\left(\frac{\partial \mathrm{Nu}}{\partial \Delta T_{s}}S_{\Delta T_{s}}\right)}^{2}\right]}^{0.5},\text{Relative error}=\frac{S_{\mathrm{Nu}}}{Nu}$$

Table 2 provide an example of Nusselt number values and uncertainty.

## 6 Hybrid nanofluids

A single-phase flow was assumed to exist between the CuO, Al_2_O_3_ nanoparticles suspended in distilled water under investigation. The nanoparticles which is selected according to the available criteria information based on reliability, efficiency, energy, economic, and environmental criteria.

**Figure 6:**
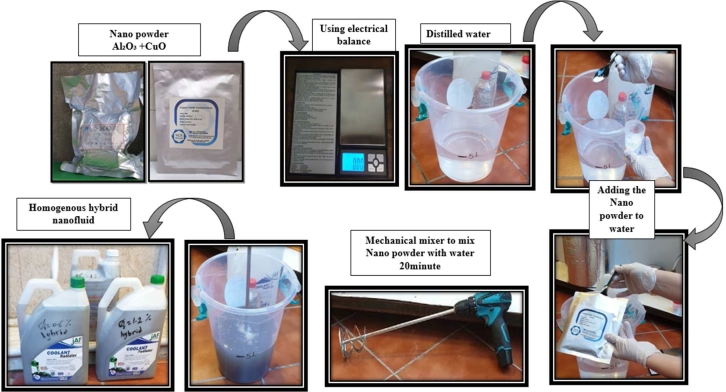
Hybrid nanofluids preparation steps.

The procedure of preparation hybrid nanofluids presented in Fig. 6, [33], [34], [35].

Volume fraction of nanoparticles in the hybrid nanofluids:

(2) $$\emptyset_{hnf}=\emptyset_{np1}+\emptyset_{np2}$$

Density of hybrid nanofluids:

(3) $$\rho_{hnf}=\rho_{np1}\emptyset_{np1}+\rho_{np2}\emptyset_{np2}+(1-\emptyset_{hnf})\rho_{bf}$$

Specific heat of hybrid nanofluids:

(4) $$Cp_{hnf}=\frac{\emptyset_{np1}\rho_{np1}Cp_{np1}+\emptyset_{np2}\rho_{np2}Cp_{np2}+{\left(1-\emptyset_{hnf}\right)}_{\rho bf}Cp_{bf}}{\rho_{hnf}}$$

Viscosity of hybrid nanofluids:

(5) $$\mu_{hnf=\frac{\mu_{bf}}{{\left(1-\emptyset_{np1}-\emptyset_{np2}\right)}^{2.5}}}$$

Thermal conductivity of hybrid nanofluids:

(6) $$K_{hnf}=K_{bf}\frac{\left(\frac{\emptyset_{p1}k_{p1}+\emptyset_{p2}k_{p2)}}{\emptyset_{hnf}}\right)+2k_{bf}+2\left(\emptyset_{p1}k_{p1}+\emptyset_{p2}k_{p2}\right)-2\emptyset_{hnf}k_{bf})}{\left(\frac{\emptyset_{p1}k_{p1}+\emptyset_{p2}k_{p2}}{\emptyset_{hnf}}\right)+2k_{bf}-\left(\emptyset_{p1}k_{p1}+\emptyset_{p2}k_{p2}\right)+\emptyset_{hnf}k_{bf})}$$

Table 3, Table 4, which list the thermal characteristics of CuO and Al_2_O_3_ nanoparticles and the characteristics of hybrid nanofluids, respectively.

**Table 3:** Thermal nanoparticles properties and base fluid [36], [37].

Properties	Distilled water	Al_2_O_3_	CuO
Density (Kg/m^3^)	997	3980	6400
Specific heat (J/kg⋅K)	4180	765	535.6
Thermal conductivity (W/m⋅K)	0.607	40	76.5
viscosity (Pa⋅s)	0.000891	x	x

**Table 4:** Hybrid nanofluids thermal properties.

∅	Density (Kg/m^3^)	Specific heat (J/kg⋅K)	Thermal conductivity (W/m⋅K)	viscosity (Pa⋅s)
0.3%	1248.28	3293.619	0.88913	0.00104
0.6%	1499.56	2704.299	1.20773	0.00123
0.9%	1750.84	2284.136	1.57035	0.00146

## 7 Data reduction

**Table:** 

Variables	Equations
• Heat flux on the outer surface around tube	Q = IV
• The local heat transfer coefficient is measured as in [38]	$h=\frac{Q}{A(T_{s}-T_{b})}$
• The mean bulk fluid temperature [39]	$T_{b}=\frac{To+Tin}{2}$
• Characteristic length	$lc=\frac{4A}{P}$
• The local Nusselt number	$Nu_{x}=\frac{h_{x}d_{h}}{K_{hnf}}$
• Effective thermal conductivity for hybrid nanofluids [40]	$Nu_{l}=-\frac{K_{eff}}{K_{bf}}{\left(\frac{\partial T}{\partial x}\right)}_{inner\;wall}$
• Reynolds Number [41]	$Re_{hnf}=\frac{\rho_{hnf\;u_{i}d_{i}}}{\mu_{hnf}}$
• Friction Factor [41]	$f=\frac{2\Delta pD}{l\rho U_{2}^{2}}$
• The thermal performance factor (TPF) [41]	$\mathrm{TPF}=\frac{\frac{Nu\;enhancement}{Nu\;without\;enhancement}}{{\left(\frac{f\;enhancement}{f\;without\;enhancement}\right)}^{1/3}}$

## 8 Numerical method

### 8.1 Geometry model

**Figure 7:**
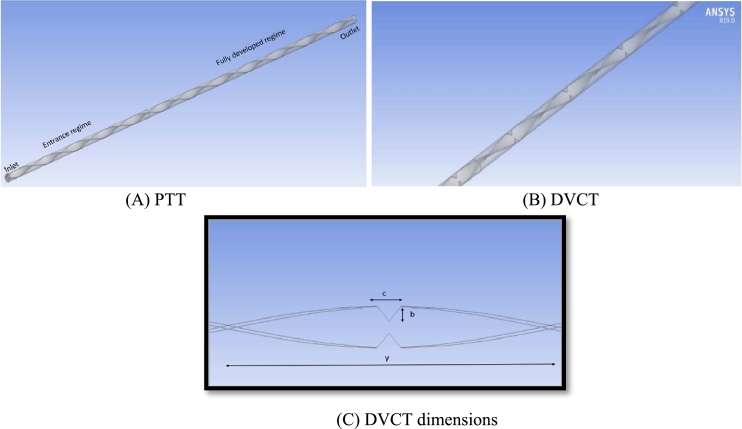
Test pipe with twisted tape.

The geometry of the tube inserted with plain twisted tape (PTT) and Double v-cut twisted tape (DVCT) are presented in Fig. 7- A, B. The current case has considered a horizontal cylindrical tube with a diameter of (0.02) m, tube length of (2) m, and constant heat flux. The heat exchanger is enhanced with inserted twisted tapes are inserted with (TR = 9.25) The design parameters of the DVCT are (1.5) mm thickness, (18) mm wide, v-cut width (c = 14.58), (b =6.14) mm v-cut depth, also (width ratio = C/y (0.088) mm and (depth ratio) = b/w (0.341) mm as shown in Fig. 7-C. The pitch is the distance that the tape must travel when the spun is 180°; the pitch length is (y = 166.6 mm) where, TR = y/W [36]. The twisted tape is made from PLA (poly lactic acid). The temperature of the hybrid nanofluids at inlet is (298k).

### 8.2 Governing equations and formulation

The governing equations of the steady-state turbulent fluid flow for the current study represented as [42].

(7) $$\frac{\partial (u_{i})}{\partial x_{i}}=0.$$

(8) $$\frac{\partial }{\partial x_{j}}\left(\rho_{hnf}u_{i}u_{j}\right)=-\frac{\partial p}{\partial x_{i}}+\frac{\partial }{\partial x_{j}}+\left(\left(\frac{\partial u_{i}}{\partial x_{j}}+\frac{\partial u_{j}}{\partial x_{i}}\right)\mu_{hnf}\right)+\frac{\partial }{\partial x_{j}}(-\rho_{hnf}\bar{u_{j}^{\prime }u_{j}^{\prime })}$$

(9) $$\frac{\partial }{\partial x_{i}}\left(\rho_{hnf}Tu_{i}\right)=\frac{\partial }{\partial x_{i}}\left(\left(\beta +\beta \right)\frac{\partial T}{\partial x_{i}}\right),\;\beta (=\mu_{hnf}/Pr_{hnf}),\;\beta_{t}(=\mu_{t}/Pr_{t})$$

In equation related to regime of fluid

(10) $$\mu_{t}=\rho_{hnf}k^{2}\frac{1}{\varepsilon }c_{\mu }$$

$$-\frac{2}{3}\delta_{ij}\frac{\partial u_{k}}{\partial x_{k}}\mu_{t}-\frac{2}{3}k\delta_{ij}\rho_{hnf}+\left(\frac{\partial u_{i}}{\partial x_{j}}+\frac{\partial u_{j}}{\partial x_{i}}\right)\mu_{t}=(-\bar{u_{i}^{\prime }u_{j}^{\prime }\rho_{hnf})}$$

and for estimating their values in nodes, RNG k – *ε* techniques

(11) $$\frac{\partial }{\partial x_{i}}\left(u_{i}\rho_{hnf}\varepsilon \right)=\frac{\varepsilon }{k}G_{k}C_{1\varepsilon }-\rho_{hnf}\frac{\varepsilon^{2}}{k}C_{2\varepsilon }+\frac{\partial }{\partial x_{j}}\left(\left(\mu_{hnf}\frac{\partial \varepsilon }{\partial x_{j}}+\frac{\partial \varepsilon }{\partial x_{j}}\mu_{t}/\sigma_{\varepsilon }\right)\right)$$

$$\frac{\partial }{\partial x_{i}}\left(\frac{\partial k}{\partial x_{i}}\left(\frac{\mu_{t}}{\sigma_{k}}+\mu_{hnf}\right)\right)-\rho_{hnf}\varepsilon +G_{k}=\frac{\partial }{\partial x_{i}}\left(k\rho_{hnf}u_{i}\right),\;C_{1\varepsilon }=1.42,C_{\mu }=0.0845,\;G_{k}=-\frac{\partial u_{j}}{\partial x_{i}}\bar{u_{j}^{\prime }u_{i}^{\prime }\rho_{hnf})},\;C_{2\varepsilon }=1.68$$

$$Pr_{t}=0.85,\;\sigma_{k}=1,\sigma_{\varepsilon }=1.3$$

### 8.3 Boundary conditions

CuO + Al_2_O_3_ hybrid nanofluids have volume concentrations of (0.3, 0.6, and 0.9%) and have an intensity of (5%), with an inlet temperature of (25 °C). CFD simulations were run with an outlet zone pressure outlet condition and a uniform input velocity at the entrance. The assumption was made that the tube's walls were flawlessly smooth, and the Re was changed between (3560 - 8320).

### 8.4 Grid generation and grid independency

**Figure 8:**
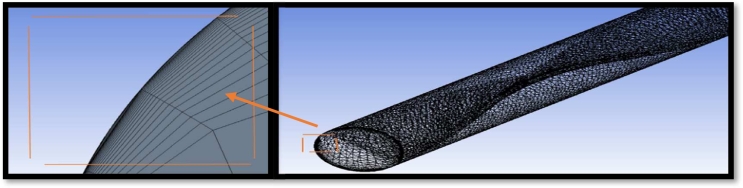
Mesh generation for present case study.

Fig. 8 depicts the computational zone employed in the numerical solution. As observed, an inflated mesh is used near the walls to capture the turbulent fluid flow's turbulent viscous sublayer effects and decrease the y+ values as possible as. Finite volume technique applied using Ansys Fluent 19.0 software. The momentum and the energy equations are discretized by using the second-order upwind scheme. The complicated geometries benefit from this. The current study considers a variety of mesh densities in order to evaluate the grid independency. To ensure grid independence, Nusselt number were recorded and sorted in each case. The numbers of nodes and elements were (859625) and (1976913), respectively, with an error of approximately 0.754%., as shown in Table 5. The convergence criteria for the governing equations are not less than $10^{-6}$.

**Table 5:** Grid dependency.

Max. face size	Element no.	Node no.	*Nu*_*ave*_
4 mm	552202	25078	35.6931
3 mm	784248	355993	36.9544
2 mm	1976913	859625	37.2354
1 mm	9107131	3656630	37.236

## 9 Validation test

**Figure 9:**
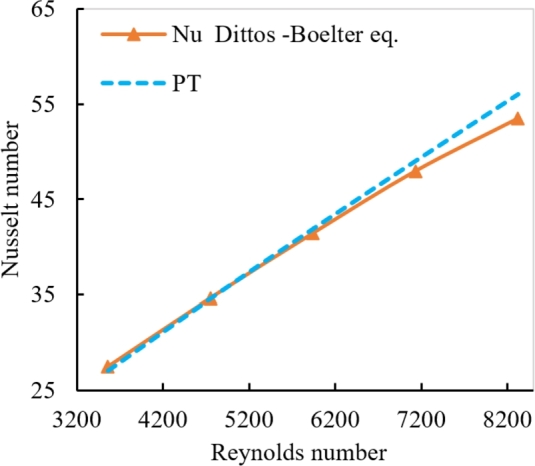
Comparison between numerical Nusselt number of distilled water with correlation of Dittus–Bolter equation.

The test section's heat transfer and pressure drop were examined in order to verify the test facilities and the values acquired. The well-known Dittus-Boelter equation, the Nusselt number, and the Reynolds number for a fully developed condition within a part using (water) are used in the current test, is shown in Fig. 9, [43].

(12) $$Nu=0.023Re^{0.8}Pr^{0.3}$$

**Figure 10:**
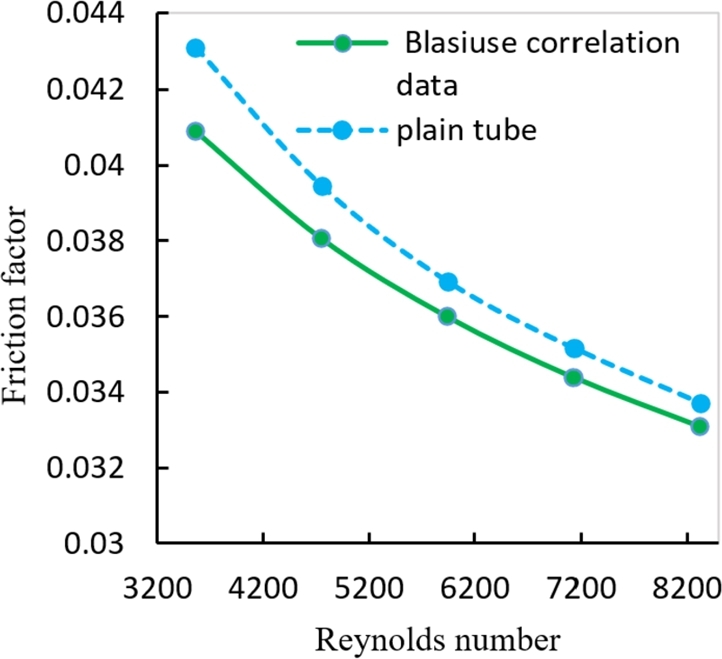
Numerical friction factor of distilled water in comparison with correlation of Blasius equation.

The results of this validation are in a good agreement between the numerical data and the data from the above correlation equation with the maximum variation of (4.638%). Agreement between the friction factor and the data from Blasius eq. (13), with a maximum divergence of (5.122%) as shown in Fig. 10.

(13) $$f=0.316Re^{-0.25}$$

## 10 Results and discussion

In this part, the numerical and experimental results of Nusselt number, friction factor, and TPF in a tube having PTT and DVCT being presented as follows:

### 10.1 Thermal effect of hybrid nanofluids with and without twisted-tape

**Figure 11:**
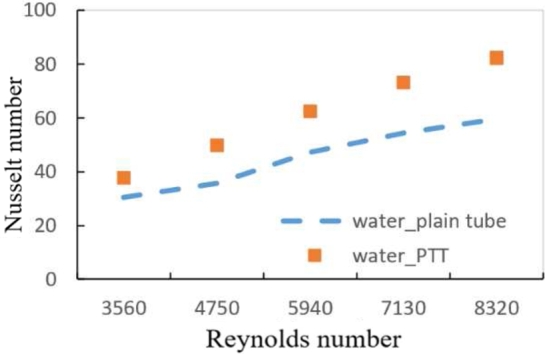
Variation of Nusselt number with Reynolds number for PTT and plain tube using distilled water.

Fig. 11 illustrates the differences between the plain twisted tape within the tube and the plain pipe with water for the volume flow rate range (3, 4, 5, 6, and 7 l/min). For the tube with a twisted insert, the Nusselt number is (37.658 and 82.418) and for the volume flow rate (3 and 7 l/min), it is (25.52 and 43.53). When compared to a horizontal tube heat exchanger, the results showed a striking rise in Nusselt number for maximum value (47.17%) and minimum value (32.28%), indicating that a plain twisted tape heat exchanger is more efficient. This behavior is caused by fluid moving in axial flow through a straight pipe with twisted tape; the swirling motion enhances flow recirculation between the tube's walls and center. On the other hand, at a particular distance from the tube length, the flow travels in a longer direction. Additionally, created secondary flow improves heat transmission, particularly at high Reynolds numbers.

Figure 12A, Figure 12B shows the relation between Nusselt number and Reynolds number for a tube with plain twisted tape with hybrid nanofluids at (0.6%, 1.2% and 1.8%) respectively. For all Reynolds number ranges, it is shown that as the Reynolds number rises, the average Nusselt number raises as well. At a (1.8%) volume fraction with hybrid nanofluid and PTT inside the tube, the data shows a (43.4%) increases in Nusselt number than hybrid nanofluids in plain tube.

**Figure 12A:**
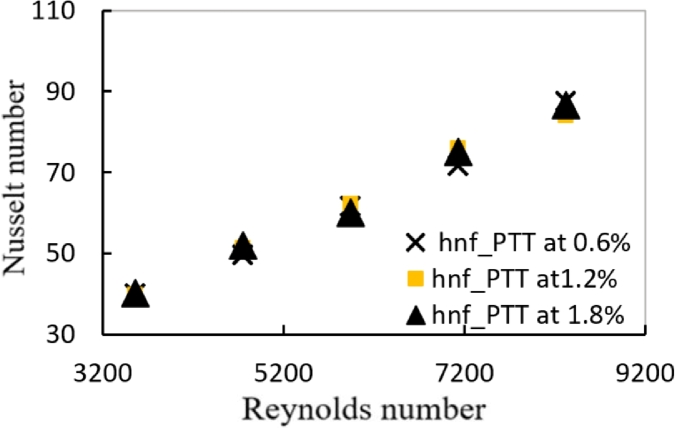
Variation of Nusselt number with Reynolds number for hybrid nanofluids and different volume fractions with PTT.

**Figure 12B:**
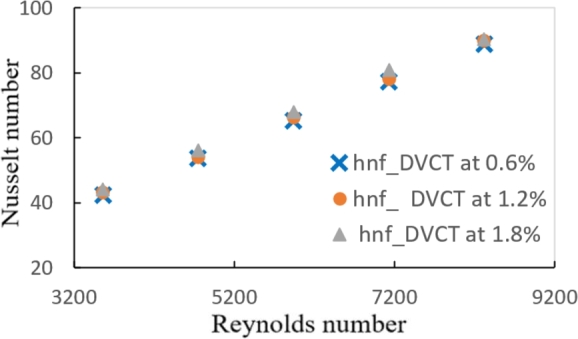
Variation of Nusselt number with Reynolds number for hybrid nanofluids and different volume fractions with DVCT.

**Figure 13:**
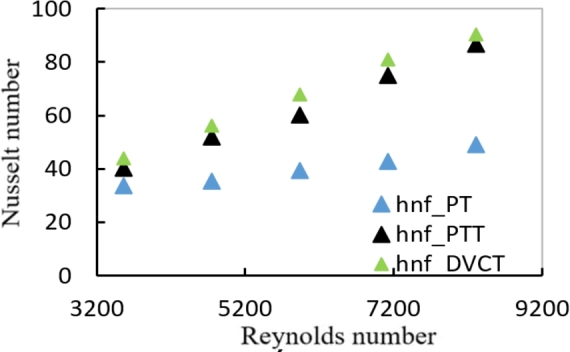
Variation of Nusselt number with Reynolds number for hybrid nanofluids at ∅ = 1.8% with different twisted tape geometries.

For PT, PTT, and DVCT tape, Fig. 13 shows the change in Nusselt number with Reynolds number. It is demonstrated for all Reynolds number ranges that the average Nu number increases along with the Reynolds number. When DVCT was inserted instead of PT tape, a maximum increase in Nusselt number of roughly (45.86%) was achieved, yielding a high Nusselt number of (1.8%).

### 10.2 Friction factor

**Figure 14:**
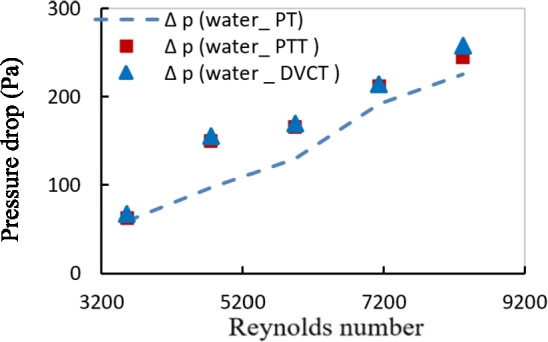
Variation of pressure drop with Reynolds number for different twisted geometries using distilled water.

**Figure 15:**
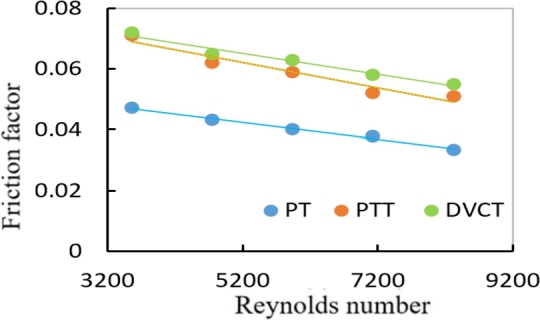
Variation of friction factor with Reynolds number for different twisted geometries using distilled water.

The pressure drop was measured to investigate the flow characteristics for the plain tube and the tube with two types of twisted tapes in fully developed region through a test tube as shown in Fig. 14. The variation of friction factor with Reynolds number for plain tube (PT) in comparison with the two types is shown in Fig. 15. The friction losses along with the circular tube increase due to the increase in the turbulent intensity of the flow that accelerates nanoparticle motion through the swirling flow, which enhancement shear stress forces near the inner tube wall. These effects on friction losses increase as nanoparticle volume concentration is increased.

**Figure 16:**
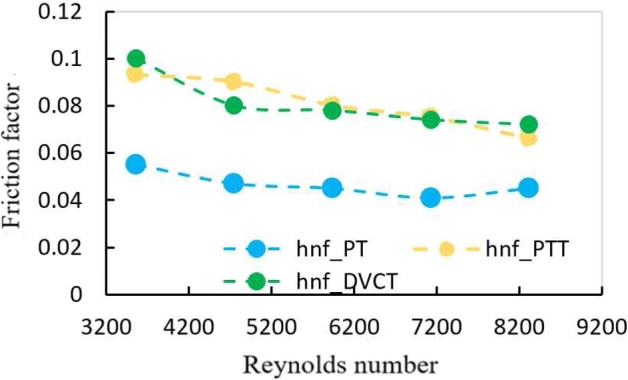
Effect of the friction factor with Reynolds Number in different geometries at (1.8%) volume fraction.

Fig. 16 shows the friction factor change for the Reynolds number for two kinds of twisted tapes with twisted ratio (TR = 9.25) at ∅=1.8%. It's observed that the friction factor has a tendency to reduce unceasingly with the rising of Reynolds number, because of the increased turbulence in the flow caused by the cuttings. As a result, the flow path between axial and swirl flows is instantly modified. The longer flow path between the walls of tube and the zone of core in comparison to the plain tube is another major cause of the higher flow resistance. It's also worth noting that the friction can be increased by the contact between fluid flow and PTT surfaces, as well as by the contact between swirl flow and tube wall. When compared to the PTT (b/c = 0), the friction factor of the hybrid nanofluid flows through heat exchanger tube with a double V-cut is raises to 31.2%.

### 10.3 Thermal performance factor (TPF)

**Figure 17:**
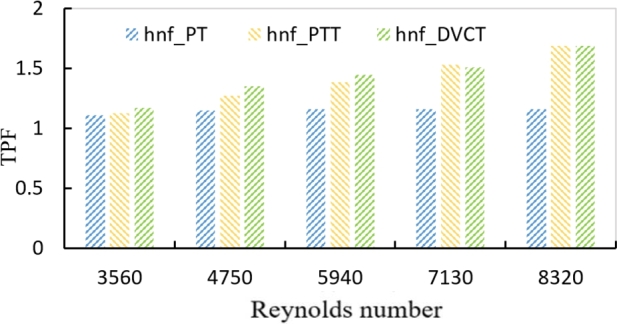
Effect of Reynolds number and different twisted geometries on TPF for hybrid nanofluids at (∅ = 1.8%).

One of the important characteristics required to describe the heat transfer enhancement is the heat transfer enhancement index, often known as the thermal performance factor. In general, a TPF greater than one reveals that the effect of the heat transfer enhancement caused by the tabulator effect of growing friction. The maximum numerical TPF with hybrid nanofluids at (1.8%) for the plain twisted tape was (1.336), and for the DVCT, it was (1.37) as shown in Fig. 17.

In Figure 18A, Figure 18B, it's worth noting that the TPF values for twisted tape inserts are greater than those for the hybrid nanofluids approach in smooth tubes. Also, as seen in the figures, the TPF values rise as the Reynold number rises, due to a large increase in the heat transfer rate created by the twisted tape. The highest value for the TPF during the present study is about (1.682%) for PTT tape and (1.69%) for DVCT at Reynold number 8320 at a volume concentration of 1.8%.

**Figure 18A:**
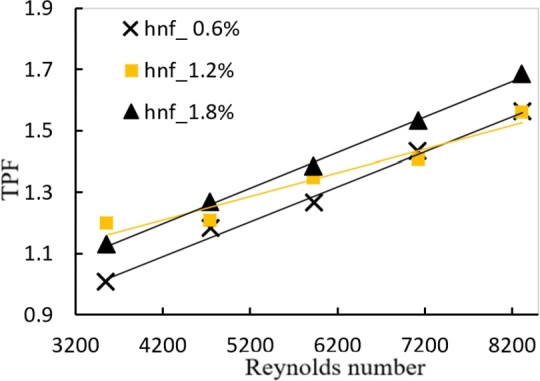
Effect of Reynolds number and volume concentration of PTT on TPF with hybrid nanofluids.

**Figure 18B:**
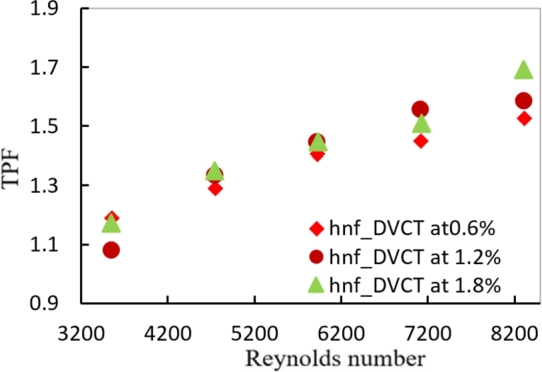
Effect of Reynolds number and volume concentration of DVCT on TPF with hybrid nanofluids.

**Figure 19:**
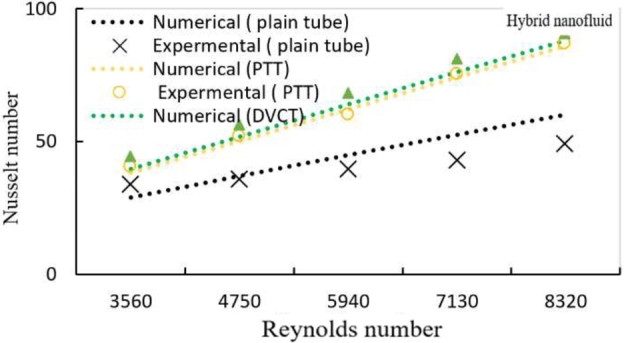
Comparison of numerical and experimental results for hybrid nanofluids at ∅ = 1.8% for different twisted geometries.

The comparison between the experimental results and numerical results is shown in Fig. 19 for the hybrid nanofluids and two types for twisted tapes at, (∅=1.8%). Good agreement between the results is noticed, and the maximum error was (9.7) % occurred with hybrid nanofluids (DVCT) tape and ∅=1.8%.

### 10.4 Temperature contours

**Figure 20:**
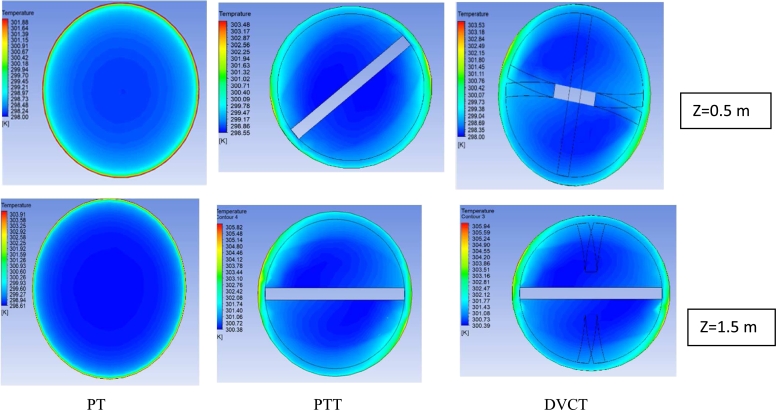
Temperature contours at different location along the test section for hybrid nanofluids (1.8%) with discharge = 7 L/min.

Fig. 20 display the contours of temperature distribution across the tube at location z = (0.5) m for volume concentration (∅=1.8%) with Re number (8320) of hybrid nanofluids, without and with two twisted-tapes inserts, respectively.

**Figure 21:**
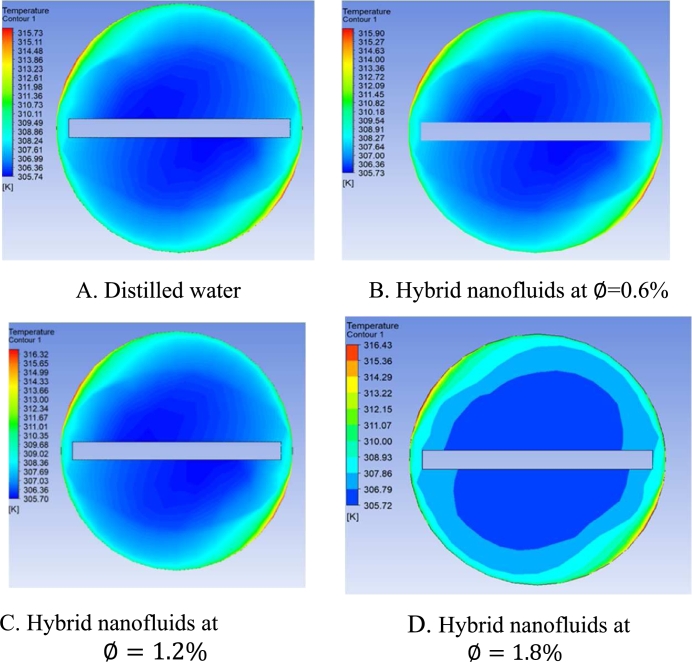
Temperature contours for different concentrations at exit of the test section for DVCT tape with (Re = 3560).

Fig. 21 shows the temperature contours for distilled water as shown in Fig. 21-A and different concentrations (∅=0.6%, 1.2% and 1.8%) for hybrid Nano fluid at the exit of the test section for double v- cut twisted tape with Re = 3560 as shown in Fig. 21-B, C, D, where the temperature increases with increasing volume concentration which increases the number of particles that increase the effective thermal conductivity (k) of hybrid nanofluid, therefore the molecular heat diffusion is augmented.

### 10.5 Empirical correlations

The gain data collection for Nusselt number and friction factor are regarding with the range for (Re, Pr, ∅ and TR) for the two types of twisted tapes and hybrid nanofluids through the following correlations as shown in Figure 22A, Figure 22B.

(14) $$Nu=aRe^{b}Pr^{c}{\left(1+\emptyset \right)}^{d}$$

(15) $$f=a_{1}Re^{b1}{\left(1+\emptyset \right)}^{c1}$$

For example, PTT at ∅=1.2%

$$Nu=3.49Re^{0.88}Pr^{-1.606}{\left(1+\emptyset \right)}^{-2.64}$$

$$f=0.449Re^{-0.47}{\left(1+1.2\text{\%}\right)}^{-0.054}$$

And DVCT at ∅=1.2%

$$Nu=2.2Re^{0.87}Pr^{-1.308}{\left(1+1.2\text{\%}\right)}^{-2.83}$$

$$f=0.314Re^{-0.403}{\left(1+1.2\text{\%}\right)}^{-0.85}$$

**Figure 22A:**
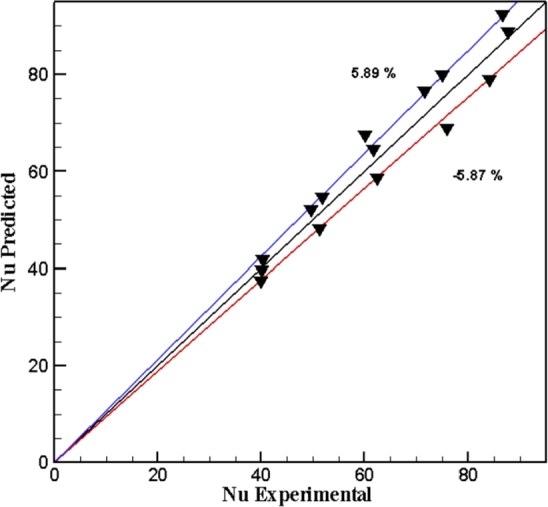
Comparison of regression equation of Nusselt number with experimental data for hybrid nanofluid in PTT.

**Figure 22B:**
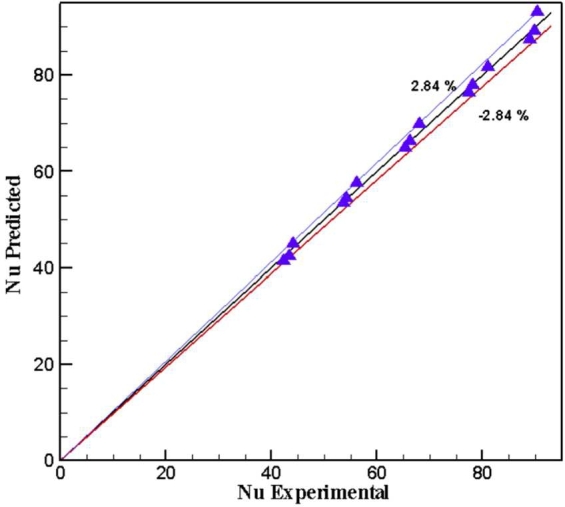
Comparison of regression equation of Nusselt number with experimental data for hybrid nanofluid with DVCT.

For (Re = 3560-8320), (Pr. = 0.6-5.243), hybrid nanofluids concentration (∅=0.6%,1.2%,1.8%) and twisted ratio (TR = 0 and 9.25).

### 10.6 Comparison with the published work

**Figure 23:**
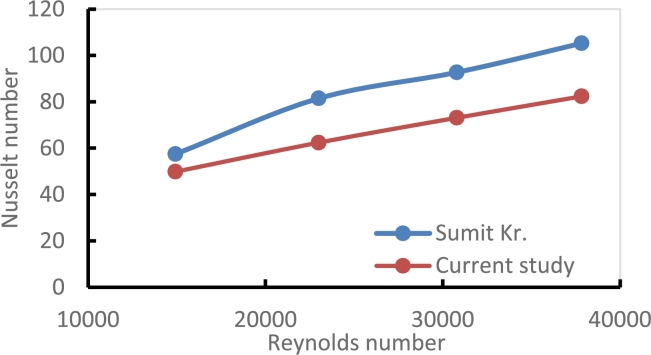
Comparison of present experimental results with the published study of ref. [44].

The comparison of the present experimental results with the published work, given in [42], is shown in Fig. 23 for Nusselt number versus Reynold number The maximum and minimum deviations were (22.7% and 13%) respectively. The deviation in the experimental results is related to the differences in the length and diameter of the test section besides some differences in nanofluids types and twisted geometries the experimental conditions and the losses associated with the experimental part for each work were not the same.

## 11 Conclusion

The present study is carried out to investigate enhancement in heat transfer characteristics by using hybrid nanofluid with and without twisted tapes. The hybrid nanofluid contains a mixture of oxide metal nanoparticles with water through a horizontal circular tube under turbulent flow with uniform heat flux, the following are the conclusions:

1. The hybrid nanofluid has higher enhancement in heat transfer compared with the base fluid at (∅=1.8%, Re = 8230) gives a higher heat transfer rate at plain tube when compared with distilled water is about (11.07%).

2. The passive method of use of (twisted tape) also increases the heat transfer enhancement, double v-cut twisted tape gives the best results than plain twisted tape when water as a working fluid enhances about (5.26%) at u = 0.317 m/s.

3. When compared to the individual use of each, hybrid nanofluid and twisted tape provide more heat transfer augmentation, with the maximum enhancement in double v-cut with hybrid nanofluid at (=1.8%, about (4.25%), CFD predict a good result for complex swirl flow with hybrid nanofluid.

4. The correlation of heat transfer, friction with PTT and DVCT were developed.

## Declarations

### Author contribution statement

Sattar Aljabair: Conceived and designed the experiments; Performed the experiments; Analyzed and interpreted the data. Noor Fouad. A. Hamza: Analyzed and interpreted the data; Contributed reagents, materials, analysis tools or data; Wrote the paper.

### Funding statement

This research did not receive any specific grant from funding agencies in the public, commercial, or not-for-profit sectors.

### Data availability statement

Data will be made available on request.

### Declaration of interests statement

The authors declare no conflict of interest.
